# Toward Inclusive Landscape Governance in Contested Landscapes: Exploring the Contribution of Participatory Tools in the Upper Suriname River Basin

**DOI:** 10.1007/s00267-021-01504-8

**Published:** 2021-08-10

**Authors:** Lisa Best, Kimberley Fung-Loy, Nafiesa Ilahibaks, Sara O. I. Ramirez-Gomez, Erika N. Speelman

**Affiliations:** 1 Tropenbos Suriname, Prof. Dr. Ruinardlaan (University Campus), CELOS Building, P.O. Box, 4194 Paramaribo, Suriname; 2 Laboratory of Geo-information Science and Remote Sensing, Wageningen University & Research, 6708 PB Wageningen, The Netherlands; 3 Department of Sustainable Management of Natural Resources, Anton de Kom University of Suriname, P.O. Box, 9212 Paramaribo, Suriname

**Keywords:** Geo-information tools, Indigenous and tribal communities, Inclusive governance, Stakeholder participation, Suriname

## Abstract

Nowadays, tropical forest landscapes are commonly characterized by a multitude of interacting institutions and actors with competing land-use interests. In these settings, indigenous and tribal communities are often marginalized in landscape-level decision making. Inclusive landscape governance inherently integrates diverse knowledge systems, including those of indigenous and tribal communities. Increasingly, geo-information tools are recognized as appropriate tools to integrate diverse interests and legitimize the voices, values, and knowledge of indigenous and tribal communities in landscape governance. In this paper, we present the contribution of the integrated application of three participatory geo-information tools to inclusive landscape governance in the Upper Suriname River Basin in Suriname: (i) Participatory 3-Dimensional Modelling, (ii) the *Trade-off!* game, and (iii) participatory scenario planning. The participatory 3-dimensional modelling enabled easy participation of community members, documentation of traditional, tacit knowledge and social learning. The *Trade-off!* game stimulated capacity building and understanding of land-use trade-offs. The participatory scenario planning exercise helped landscape actors to reflect on their own and others’ desired futures while building consensus. Our results emphasize the importance of systematically considering tool attributes and key factors, such as facilitation, for participatory geo-information tools to be optimally used and fit with local contexts. The results also show how combining the tools helped to build momentum and led to diverse yet complementary insights, thereby demonstrating the benefits of integrating multiple tools to address inclusive landscape governance issues.

## Introduction

Nowadays, a multitude of formal and informal institutions, public and private organizations, and local actors pursue competing interests in space and/or time in increasingly complex multifunctional tropical forest landscapes (Sayer et al. 2013; Kusters et al. 2020). While tropical forests harbor rich biodiversity and play a key role in mitigating climate change, they are also home to many indigenous and tribal communities, who manage and depend on the forest for their livelihood (Byron and Arnold 1999; Chao 2012). The communities’ traditional lifestyles and governance structures are based on a strong relationship with the surrounding natural environment and a strong sense of place (van Opstal and Hugé 2013; Hill et al. 2020). Post-colonial developments exposed indigenous and tribal communities to the influences of modern technologies, market economics, and political dynamics. These influences result in external claims on natural resources within the communities’ territory and changes in their traditional ways and relationship with the forest (Byron and Arnold 1999; Groenfeldt 2003; Gómez-Baggethun et al. 2010; Reyes-García et al. 2013).

Due to a history of marginalization and, in cases, lack of formal land tenure rights, indigenous and tribal communities often have limited legal protection and are in a disadvantaged position from the onset (Simpson 2004). Commonly, these communities have little influence on formal landscape management decisions (Timoti et al. 2017; Kusters et al. 2020), and are up against powerful private and public actors (Reed et al. 2020). At the same time, communities are disempowered to participate, share knowledge and voice their concerns (Evans et al. 2008; Ban et al. 2013). In these contested landscapes complex problems arise concerning local and non-local stakeholders. As such, sustainable management requires coordination of multi-level decision making (Arts et al. 2017). Moreover, it requires reconciling competing interests, sound land-use planning, cross-sectoral coordination, and inclusive governance mechanisms (Kozar et al. 2014; Sayer et al. 2015; Kusters et al. 2020). Unfortunately, mechanisms to facilitate meaningful participation of stakeholders across sectors and scales are often still lacking.

The concept of landscape governance emerged in response to address these challenges, touching upon socio-cultural, economic, and political dimensions and ecological functioning (Görg 2007; Sayer et al. 2013). We define landscape governance as multi-level decision making and the set of rules on the natural conditions of places and socially constructed spaces in the landscape (Kozar et al. 2014; Ros-Tonen et al. 2014; Arts et al. 2017; Kusters et al. 2020). There is no single definition of what comprises good landscape governance, but there appears to be some agreement on important criteria (McCall and Dunn 2012; Kozar et al. 2014; Kusters et al. 2020). These include: (i) accountability, (ii) transparency, (iii) equity, (iv) collaboration, (v) coordination, and (vi) inclusiveness.

Governance in tropical forest landscapes where indigenous and tribal communities reside involves formal and “informal”, customary institutions. Indigenous and tribal communities have traditional knowledge, often not formally documented on paper, that can be invaluable to landscape decision making and policies (Pfeffer et al. 2013; McGonigle et al. 2020; Needham et al. 2020). This traditional, tacit knowledge is embedded in social-ecological spatial relationships (McCall and Dunn 2012; Pfeffer et al. 2013; Akbar et al. 2020) and is passed down through generations (Gadgil et al. 1993). Despite the importance of traditional knowledge, recognition of local values, knowledge, and customary institutions is lacking in formal governance processes (Kozar et al. 2014; Lyver and Tylianakis 2017; Timoti et al. 2017; Needham et al. 2020). Inclusive landscape governance thus goes beyond participation in decision making and rather concerns meaningfully bringing together diverse landscape actors with different values and interests, legitimizing voices, knowledge systems, and institutions of marginalized communities and balancing power dynamics (Kozar et al. 2014; Arts et al. 2017).

The use of geo-information tools has become more prominent in conservation and natural resource management (Görg 2007; Sayer et al. 2013; Brown and Fagerholm 2015). Using culturally appropriate, participatory geo-information tools, i.e., respecting the customary structures and considering education levels, language barriers, and traditional gender roles, presents opportunities to stimulate more inclusive processes. For example, by integrating different knowledge systems and enabling low-literate persons to participate in landscape governance discussions (Sheil et al. 2002; McCall and Dunn 2012; Pfeffer et al. 2013; De Haan 2016; Akbar et al. 2020). Tools, varying from very simple to very complex, have been developed over the years, such as Participatory Rural Appraisal and Rapid Rural Appraisal (Chambers 1994), Participatory Geographic Information Systems including participatory mapping using sketch drawings, participatory 3D modelling, open source satellite imagery, and geographically linked mobile applications (Harris and Weiner 1998; Brown and Kyttä 2018), more sophisticated tools such as interactive map tables and interactive web-based cartography tools (Flacke et al. 2020), and simulation tools such as serious games (Speelman 2014; Meinzen-Dick et al. 2018).

In this paper, we present the contribution of the integrated application of three participatory geo-information tools to inclusive landscape governance in the Upper Suriname River Basin (USRB) in Suriname, ancestral home to the Saamaka maroon tribe for centuries. First, we describe the application of three spatially explicit, participatory tools via a case study in the USRB, where we used (1) Participatory 3-Dimensional Modelling (P3DM), (2) the *Trade-off!* game, and (3) participatory scenario planning (PSP). Then, we discuss the strengths of these spatially explicit, participatory tools for landscape governance, and their combined use. We base our discussion on the criteria of landscape governance (Table 1). We look at inclusiveness, as the extent to which the tools can enhance fair participation and help to legitimize local voices and knowledge. With accountability, we look at the ability of the tools to support transparent processes and to involve different actors at all stages. In the case of equity, we look at the extent to which the tools help to empower users and have the ability to involve disadvantaged groups. With collaboration and coordination, we look at the ability of the tools to stimulate interaction between different types of landscape actors, mutual understanding, social learning, and discussions on trade-offs. In the case of competence, we look at the extent to which the tools build the capacity of its users for area management, communication, negotiation, and accessing information. Lastly, we discuss the overall added value of using integrated tools for addressing inclusive landscape governance.

**Table 1:** Framework for discussing geo-information tools’ potential to contribute to inclusive landscape governance (McCall and Dunn 2012; Kozar et al. 2014; Chung et al. 2019; Flacke et al. 2020; Kusters et al. 2020)

Elements of landscape governance	Description
Inclusiveness	Fair participation in decision making, integration of different knowledge systems and legitimacy of local tacit knowledge and informal governance systems. Relevant aspects: legitimacy, participation, ownership, local knowledge.
Accountability	Transparent decision-making processes and existence of mechanisms for landscape actors to be held accountable based on their responsibilities. Relevant aspects: transparency, actor involvement in all processes, accountability mechanisms.
Equity	Balanced power relations and levels of influence, and inclusion of disadvantaged groups. Relevant aspects: balanced power dynamics, empowerment, gender.
Collaboration and coordination	Integration of different sectors and levels, negotiation of trade-offs and balancing of social, ecological and economic outcomes. Relevant aspects: integrated landscape planning, understanding of other actors’ perspectives, social learning, negotiating trade-offs.
Competence	Building capacity building in terms of area management, communication and negotiation and accessing information. Relevant aspects: management abilities, confidence, knowledge exchange.

## Methods

### Study Area

**Fig. 1:**
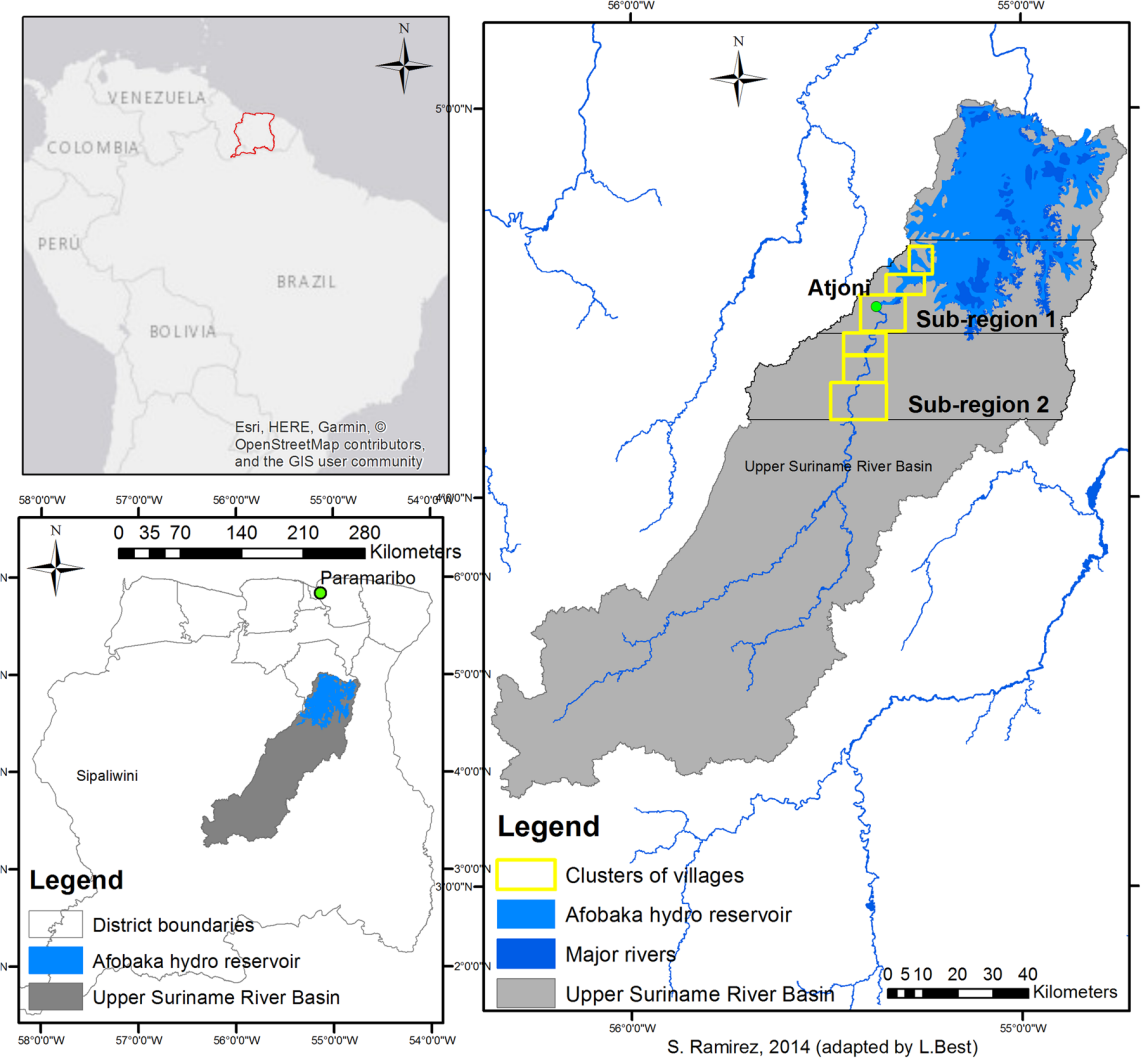
Study area: the Upper Suriname River Basin and the two subregions

The USRB lies in the center of Suriname and can be reached by road until the main landing place, Atjoni, and from there by boat (Fig. 1). The area is covered by 124,989 hectares of primary forest and 75,906 hectares of secondary forest (Ramírez-Gomez et al. 2017). The afro-descendent communities belonging to the Saamaka tribe have inhabited the USRB since the seventeenth century and currently comprise a population of around 17,954 people (General Bureau of Statistics 2012) in 62 villages spread along the river. In Suriname, indigenous and tribal communities do not have legally recognized collective land tenure rights.

### Livelihood and Land Use

The livelihood of the Saamaka is based on shifting cultivation on fields of up to 1 hectare scattered around the villages, and further depends on forest ecosystem services for basic and household needs. Other means of income generation include the collection of non-timber forest products (NTFPs) to make oils, boat transport, and employment at tourism lodges. In addition, some villages in the downstream area earn an income from third-party logging in their community forest concessions. These are concessions granted to communities by the government to support local livelihoods.

Within the USRB, we selected two study subregions (Fig. 1), based on logistic and financial feasibility, where the tools were applied. Subregion 1 is closer to Atjoni and currently has 60 km of dirt road extension. This subregion currently has 36,300 hectares in active community forest concessions, while an additional 37,424 hectares have been requested for new community forest concessions (SBB 2019). The community forest concessions are not collective land rights, but provisions in the national Forest Management Act (1992) that allow communities to use forest resources in a government-assigned area for subsistence and commercial purposes. Community forest concessions are often exploited unsustainably by third parties because the communities do not have sufficient know-how and capacity to exploit the concession themselves. This discrepancy weakens the negotiating position of the communities, resulting in sub-optimal agreements with few benefits for the community. By contrast, subregion 2 is more remote and has no road infrastructure nor granted community forest concessions, although concessions covering 42,368 hectares have been requested (SBB 2019).

### Customary Governance

The use of the Saamaka territory is governed by the customary structures in place for centuries: a chief at the head of the tribe who is responsible for the entire USRB, head captains (leaders from each of the 12 *Lo*’s or clans), captains (village leaders) and the *Basia*s (assistants of the village leaders). Historically, the Saamaka tribe consists of 12 *Lo*’s among which land was divided, and that are subdivided by the *Beë* (matrilineal families). Use of resources on another family’s piece of land, e.g., opening up an agriculture field, is allowed with permission from the *Beë* to which the land belongs. Villages were founded on land belonging to a certain *Lo*. Over the years, transmigration after the construction of the hydroelectric dam, migration, and family unions have led to the mixing of community members from different *Lo*’s and *Beë*’s over the different villages.

This customary governance system is not entirely recognized in formal land management regulations and procedures (Heemskerk 2003). Formally, the USRB is a ressort, the smallest administrative unit, within the district of Sipaliwini (Fig. 1) and is governed by the district authorities. Many public services, whether or not via extension offices at Atjoni, are still managed by the central government, situated 200 km away in the capital Paramaribo. As a consequence, decision making, planning, and development in the USRB take place in a top-down, uncoordinated manner, increasing risks of land-use conflict (Ramírez-Gomez et al. 2017).

The non-inclusive decision-making processes, lack of legitimacy of customary governance and knowledge systems, and associated power dynamics are not promoting sustainable use of the USRB. This can lead to further marginalization of the Saamaka and even conflict, as they are directly affected by land-use decisions and actions of non-local actors. A clear example is the community forest concessions, which are granted to a village belonging to a *Lo*. However, the land, ultimately assigned by the government, may be overlapping with land from another *Lo* who did not give permission or was not involved. Existing regulations attempt to include local communities in decision making (such as the procedure for community forest concessions, or the ressort hearings for the annual district development plan), but in practice, participation is selective and outcomes benefiting the communities are limited.

### Participatory Tools toward Inclusive Landscape Governance

**Fig. 2:**
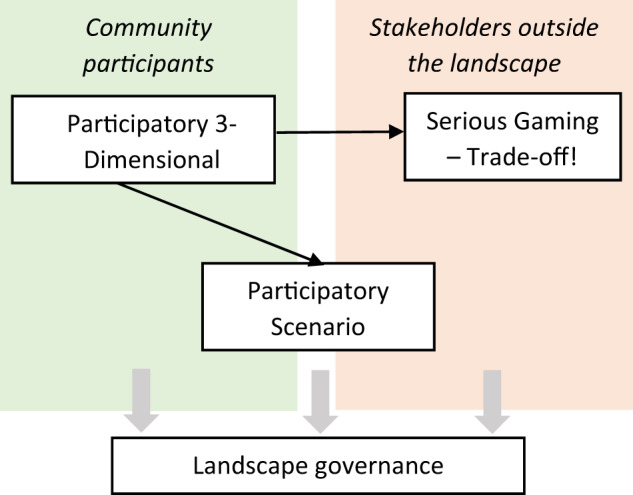
Schematic overview of spatially explicit tools applied and participant groups

The application of three spatially explicit, participatory tools in our case study was to assess the current and future supply of ecosystem services in light of sound land-use planning in the USRB. After describing the application of the tools, we assess their strengths and weaknesses in light of landscape governance. The three tools applied are P3DM, the *Trade-off!* game, and PSP (Rambaldi 2010; Addison and Ibrahim 2013; Verutes and Rosenthal 2014) (Fig. 2). The P3DM was applied in the two subregions to map the Saamaka living area and assess important ecosystem services. This tool was selected because of its simplicity and tangibility and allowed community participants to “be in the driver’s seat”. The *Trade-off!* game was applied at the landscape level to educate stakeholders on the concept of trade-offs in land-use decision making. The *Trade-off!* game is part of the Integrated Valuation of Ecosystem Services and Trade-offs (InVEST) modeling tools developed by the Natural Capital Project (Stanford University 2021). It was selected as a simple, visual tool and presented an accessible way of engaging with stakeholders. The PSP was applied to develop spatially explicit scenarios from the perspective of the local communities and non-local stakeholders. This tool includes the Scenario Generator (WWF and Natural Capital Project 2021), which is also linked to the InVEST modeling tools. The PSP was selected due to its spatially explicit visualization of future perspectives. The three tools were linked as follows: geographical information gathered during the P3DM was used to adjust the playing boards of the *Trade-off!* game to the local context of the USRB. In addition, the output of the P3DM exercise was used as the starting land cover in the PSP process. Following official approval by Saamaka traditional authorities from subregion 1 and 2, respectively, based on principles of free, prior and informed consent, P3DM was applied first.

### Participatory 3-Dimensional Modelling

P3DM is a mapping tool that utilizes location-specific social-ecological community knowledge to construct a physical, scaled model representation of the landscape. P3DM combines the co-production of tacit knowledge and existing geographic information technologies for the benefit of marginalized, resource-dependent communities (Rambaldi 2010). In our study, the aim with P3DM was to collectively map the Saamaka territory, important ecosystem services and provide the community with a self-constructed communication and negotiation tool. The approach follows several stages involving youth, women, men and elderly community members (for more information: Ramírez-Gomez et al. 2017). Our application of the P3DM tool consisted of: (1) the elucidation of the map legend; (2) construction and populating of the model; (3) digitization of the model through high-resolution photographs and georeferencing; and (4) validation of the P3DM maps by the community participants. This was a crucial last step to reinforce trust and ownership of the mapped product among the community participants. The last stage of the P3DM included an official presentation event for stakeholders from Paramaribo (policymakers, civil society organizations, private companies)^1^, followed by a brief reflection with external stakeholders regarding the perceived usefulness of P3DM in landscape governance. Additional details on the P3DM tool are found in Online Resource 1-A.

### *Trade-off!* Game

**Fig. 3:**
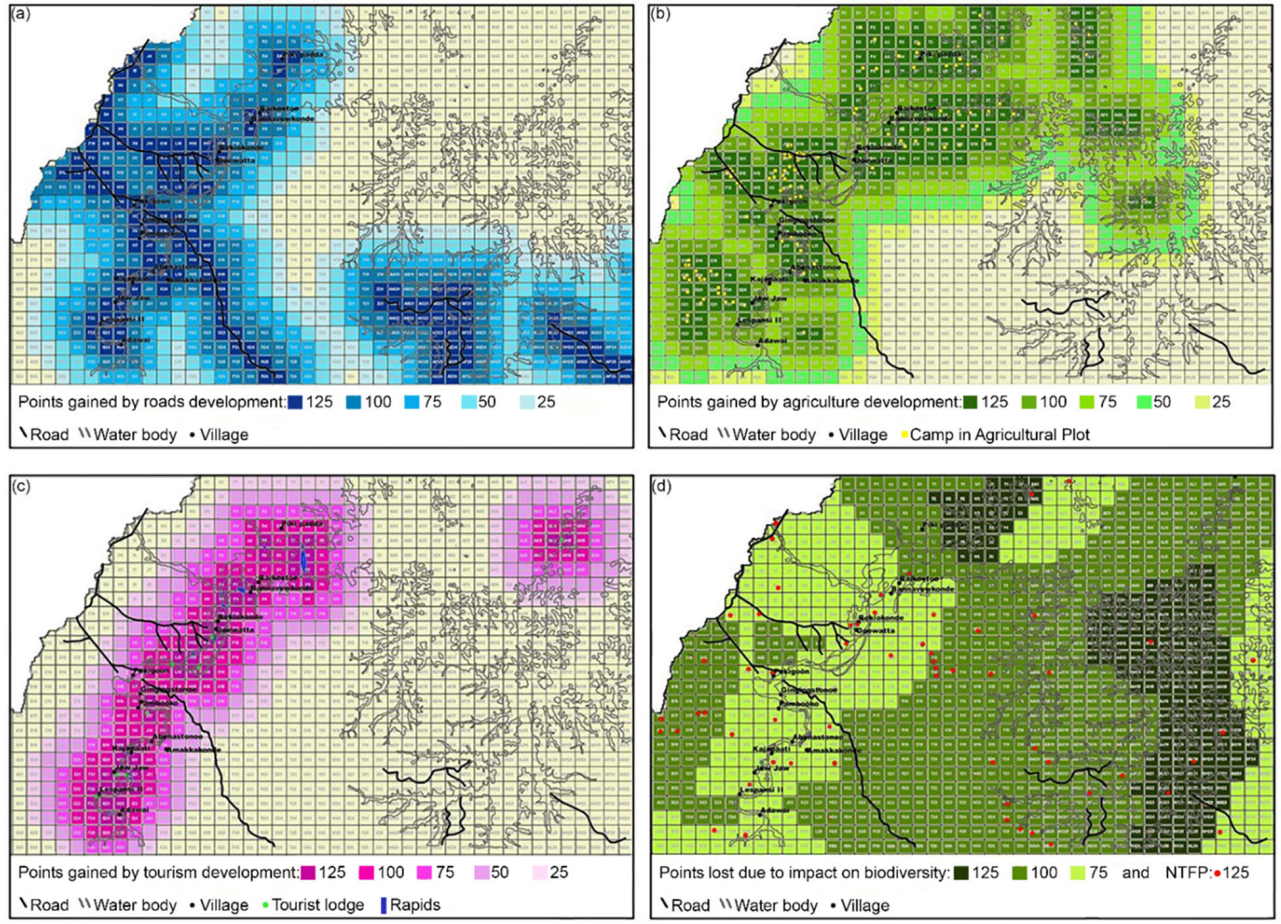
The printed board maps of the adapted *Trade-off!* game for the Upper Suriname River basin: **a** road development, **b** agriculture development, **c** tourism development, and **d** ecosystem services (biodiversity and NTFPs)

The *Trade-off!* game (^©^Natural Capital Project) is an educational game consisting of maps placed on cardboard, playing pawns and a score calculator. In our case study, the aim with the *Trade-off!* game was to introduce the concept of trade-offs and reflect on stakeholders’ land-use decisions in the USRB. The objective of the game is to educate players on the value of ecosystem services and trade-offs in land-use decisions, thereby contributing to increased awareness of the impacts of decision making in a landscape. The game is part of the InVEST approach and conceptualizes the issue of land-use trade-offs by integrating nature’s values into the land-use planning process in a simple and interactive manner (Verutes and Rosenthal 2014). Serious gaming is a relatively new approach to understand system dynamics and help stakeholders to collectively explore strategies for managing natural resources in the safety of a game setting (Speelman 2014; Meinzen-Dick et al. 2018; Van Noordwijk et al. 2020). We adapted the *Trade-off!* game to the USRB, by using digitized GIS data obtained from the P3DM to make it more relatable and increase stakeholder interaction (additional information can be found in Online Resource 1-B). The adapted game consists of four playing boards, namely: (a) road infrastructure, (b) agriculture, (c) tourism, and (d) ecosystem services (Fig. 3). The game is played within two rounds with groups of five to eight persons. In our case study, each group represented a different stakeholder type, namely (i) government (two groups), (ii) private sector, (iii) non-governmental institutions, and (iv) academic institutions. Groups were not mixed in order to determine how the results would reflect playing strategies for the different stakeholder types. The objective for the participants during each round is to obtain the most points by strategically distributing their pawns on the map. In the first round, the highest score represents the highest economic gain and wins the round. In the second round, participants have to consider points lost due to the impact of economic development on ecosystem services and improve their net score. Additional details on the *Trade-off!* game are found in Online Resource 1-B.

### Participatory Scenario Planning

**Fig. 4:**
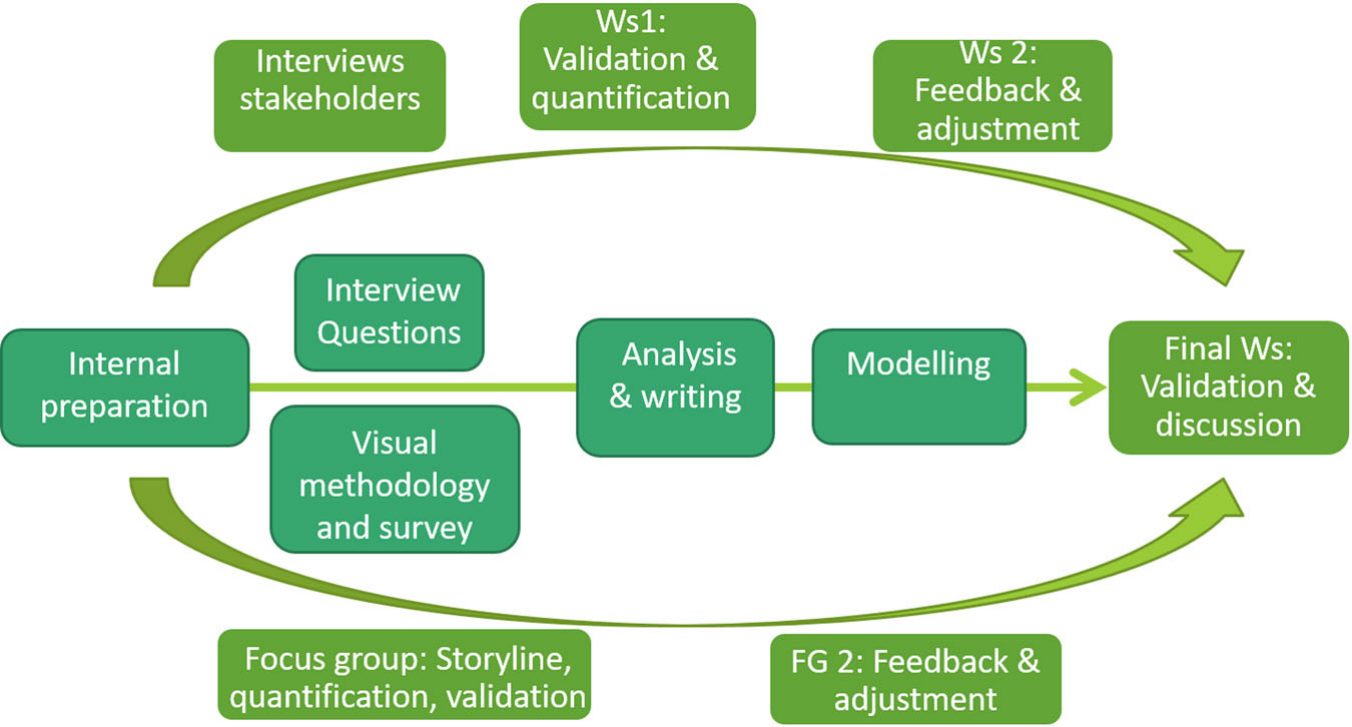
An overview of the participatory scenario planning process with participants from the Saamaka community (focus groups) and stakeholders from Paramaribo (Ws: workshops)

In our case study, the aim of the PSP was to identify an ideal, yet plausible, future for the Saamaka and the external stakeholders as a starting point for working toward reconciliation of competing interests in the landscape. PSP can be used as a strategic tool to bring actors together to envision possible future pathways, especially in cases where natural resources or land-use conflicts exist (Patel et al. 2007; Accastello et al. 2019). The spatially explicit iterative approach was adapted from (McKenzie et al. 2012; Addison and Ibrahim 2013) and based on the InVEST Scenario Generator tool (^©^Natural Capital Project). The Scenario Generator models land cover change from land cover transition probability and land suitability factors (Berg et al. 2016). It is a relatively simple tool to incorporate stakeholder input and translate this to spatially explicit scenarios, which is particularly useful when data and resource availability are limited (Ritzema et al. 2010; Carnohan et al. 2020). The different phases of the PSP exercise in the USRB consisted of (i) preparatory work, (ii) gathering information from different stakeholder groups, (iii) drafting preliminary storylines, (iv) gathering feedback from stakeholders on the storylines and adjusting accordingly, (v) finalizing scenarios, and (vi) a plenary workshop (Fig. 4).

The process of gathering participant input, drafting storylines, validation, and spatially explicit modeling was largely similar for both groups. In the case of the communities more visual materials were used to gather input, to take into consideration the literacy and education levels of participants. Even though the approaches somewhat differed between stakeholders and communities, both covered similar topics (Table 2). For the scenarios, a short timeline of 10 years was used, for the relatability of stakeholders and community participants. The last step in the process consisted of a joint workshop where the participants discussed the scenarios, similarities, and differences. Additional details on the PSP method are found in Online Resource 1-C.

**Table 2:** An overview of topics covered during interviews with stakeholders in Paramaribo and focus group discussions with the Saamaka community

Topics from semi-structured interviews	Topics covered during focus group discussions using visual aids
Interest in the landscape, ecosystem services of importance	Basic needs
Perception on drivers of change	Demography
Perception on historical changes and future development in the landscape	Land use
Trends in availability and use of ecosystem services	Dealing with “surprises” (unforeseen situations)
Management strategies and roles; including “surprise situations”	Governance and social organization
	Environmental management strategies

## Results

### Participatory 3-Dimensional Modelling

**Fig. 5:**
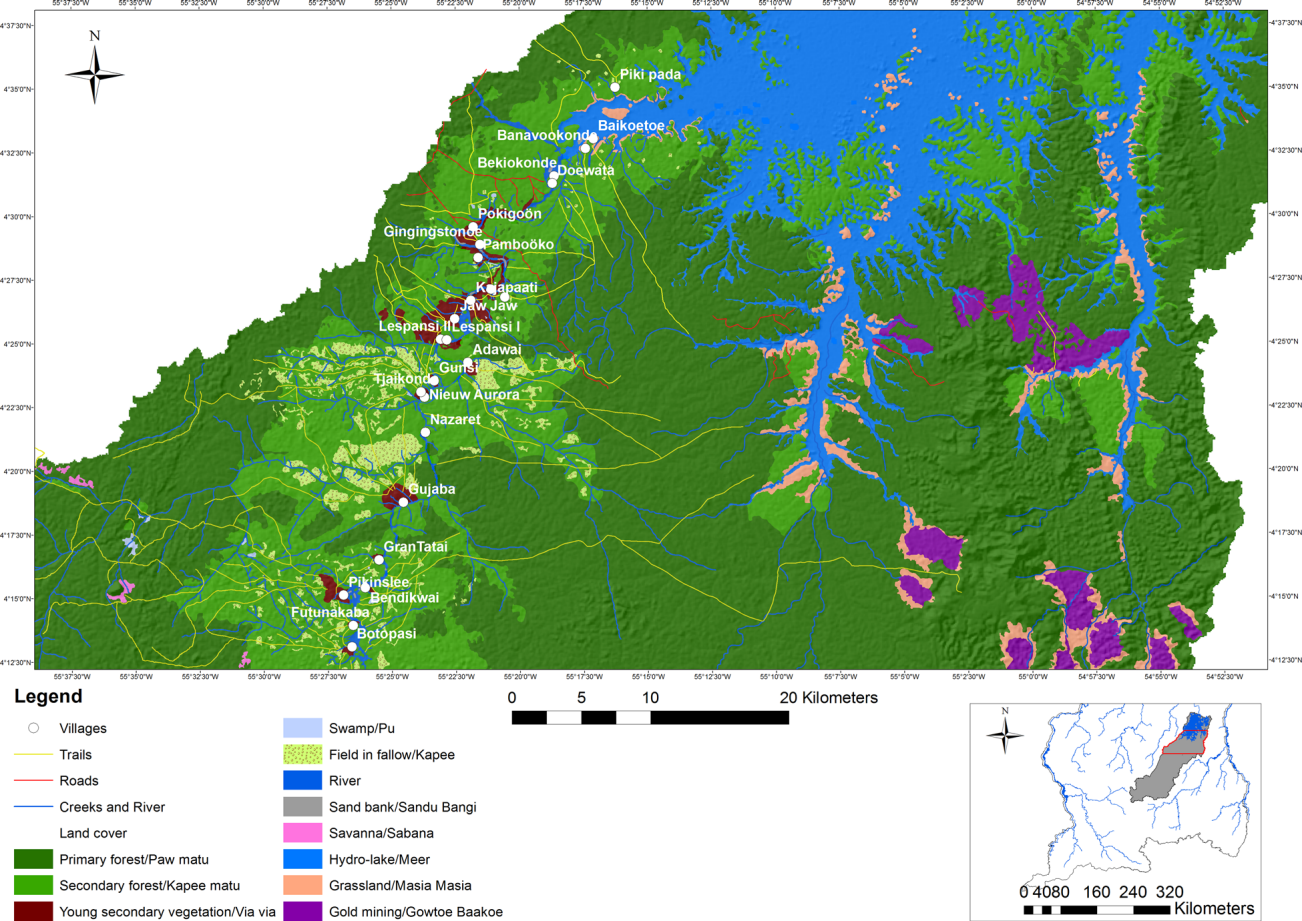
Digitized map of the study area, based on the P3DM. Note: this version of the map does not include all information that the communities placed on the map

**Fig. 6:**
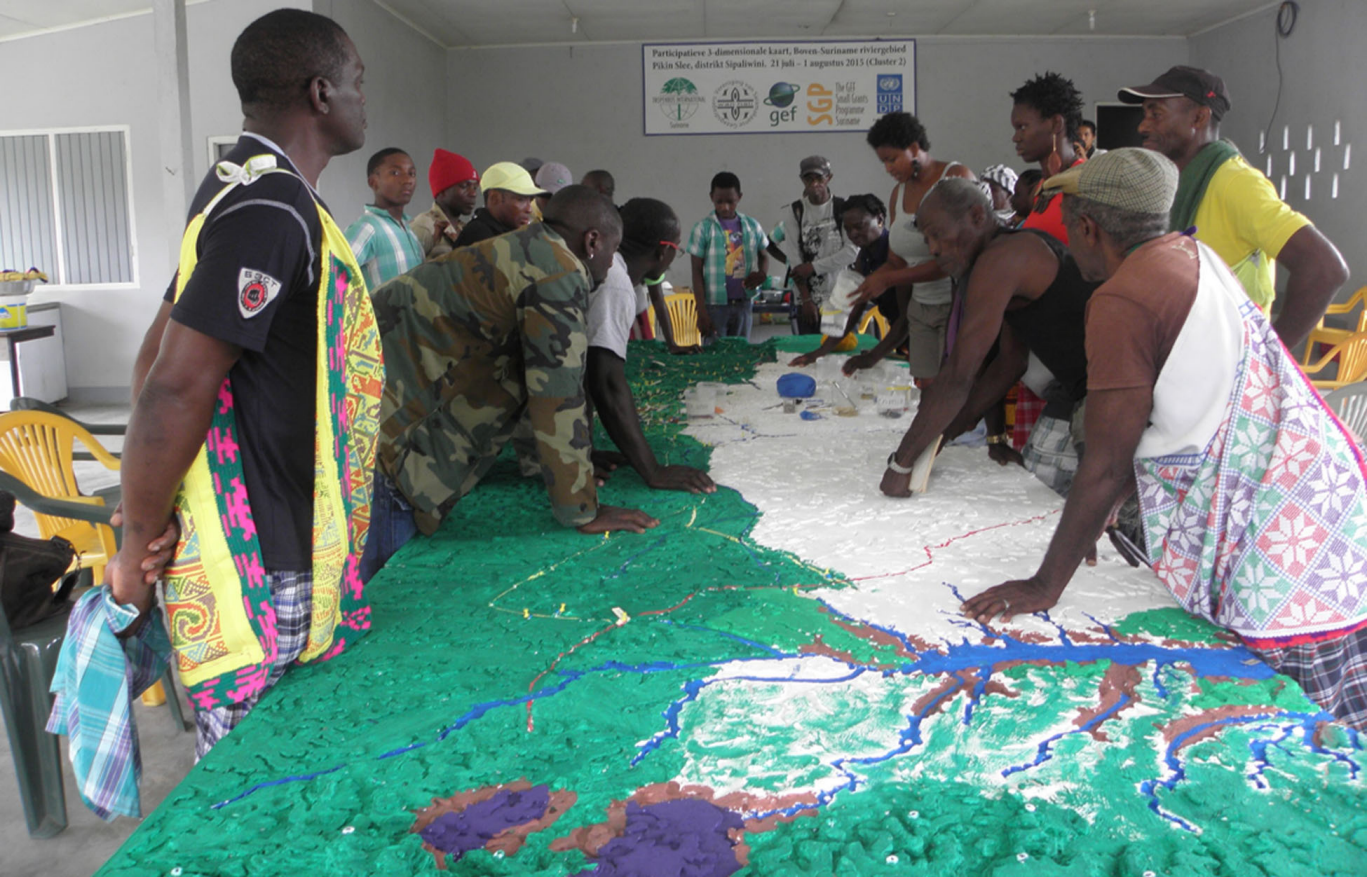
Photograph of the facilitated P3DM process. Participants are seen in discussion while populating the model

The application of the P3DM tool resulted in a physical, scaled model (Figs 5 and 6) and the identification, mapping, and prioritization of 21 ecosystem services. The physical model remains with the community as a tool to be used for various purposes. Ecosystem services mapped ranged from provisioning services such as drinking water, firewood, and forest medicines, to immaterial ecosystem services such as place identity, sacred rituals, and attachment to the territory (Table 3). Given the communities’ worldview and way of life, it was not surprising that the identified ecosystem services were mostly provisioning and cultural ecosystem services, rather than regulating and supporting ecosystem services (Haines-Young and Potschin 2011). The land covers of the P3DM represented types of ecosystems, such as primary forests, field in fallow, swamps, and rivers and creeks.

**Table 3:** Important ecosystem services identified by participants during the P3DM process

Provisioning ecosystem services important to participants	Cultural/immaterial ecosystem services
Forest medicines	Attachment to the territory
Firewood	Place identity
Resins	Sacred rituals and places
Quarry	Tourism opportunities
Fibers	Recreation
Binding and thatching materials	Transport ways
Spices	Biodiversity reservoir
Timber	Area for bathing, washing dishes and clothes, and socializing
Wild fruits	
Fish	
Palm oils	
Crops	
Wild meat	
Drinking water	

**Fig. 7:**
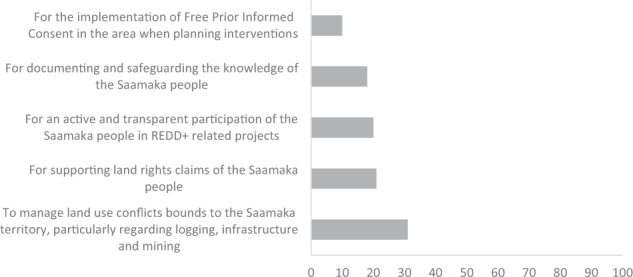
Opinion of stakeholders on the utility of the P3DM tool related to aspects of inclusive governance

At the end of the P3DM, stakeholders shared their views on the tool’s utility for inclusive governance (Fig. 7). In general, stakeholders found the P3DM tool useful to address several landscape governance issues; more than 30% of the responses indicated the utility of the P3DM tool to address land-use conflicts in the Saamaka territory. Furthermore, stakeholders found the P3DM tool mostly useful for supporting land rights claims and participation in REDD+ projects.

### *Trade-off!* Game

During the first round, all participant groups scored above 90,000 points (Table 4). Team 2, private sector, gained the most points, while team 3, government I, gained the least points. After explaining the loss of points due to environmental impact, team 2 still had the highest net score. During the second round, all groups, except government I and government II, changed their strategy by limiting their economic developments. The government II team was able to limit their environmental impact to obtain the highest net score. The government I team gained and lost the least amount of points, indicating that their strategy was more conservation motivated. Both government teams and the NGO team used a strategy where they deliberately decreased their environmental impact. Overall, all teams improved their net scores in the second round. The government II team had the highest net score in round 2 and the largest improvement from the first round. The private sector, NGO, and Academia teams had relatively little improvement during the second round, suggesting they had trouble adjusting their strategy.

**Table 4:** Scores of each team after playing the *Trade-off!* game

	Round 1			Round 2			
	Points gained	Points lost	Net score	Points gained	Points lost	Net score	Improvement
Team 1 (government I)	94,625	87,600	**7025**	95,200	78,475	**16,725**	**9700**
Team 2 (private sector)	98,450	88,825	**9625**	98,350	85,125	**13,225**	**3600**
Team 3 (government II)	91,150	90,050	**1100**	87,800	79,225	**8575**	**7475**
Team 4 (NGOs)	92,450	83,725	**8725**	82,750	73,225	**9525**	**800**
Team 5 (Academics/research)	93,875	89,500	**4375**	87,450	79,900	**7550**	**3175**

The bold scores represent the net scores which are calculated from the points gained and points lost. These represent the scores which determined who won and who lost during each round

### Participatory Scenario Planning

**Fig. 8:**
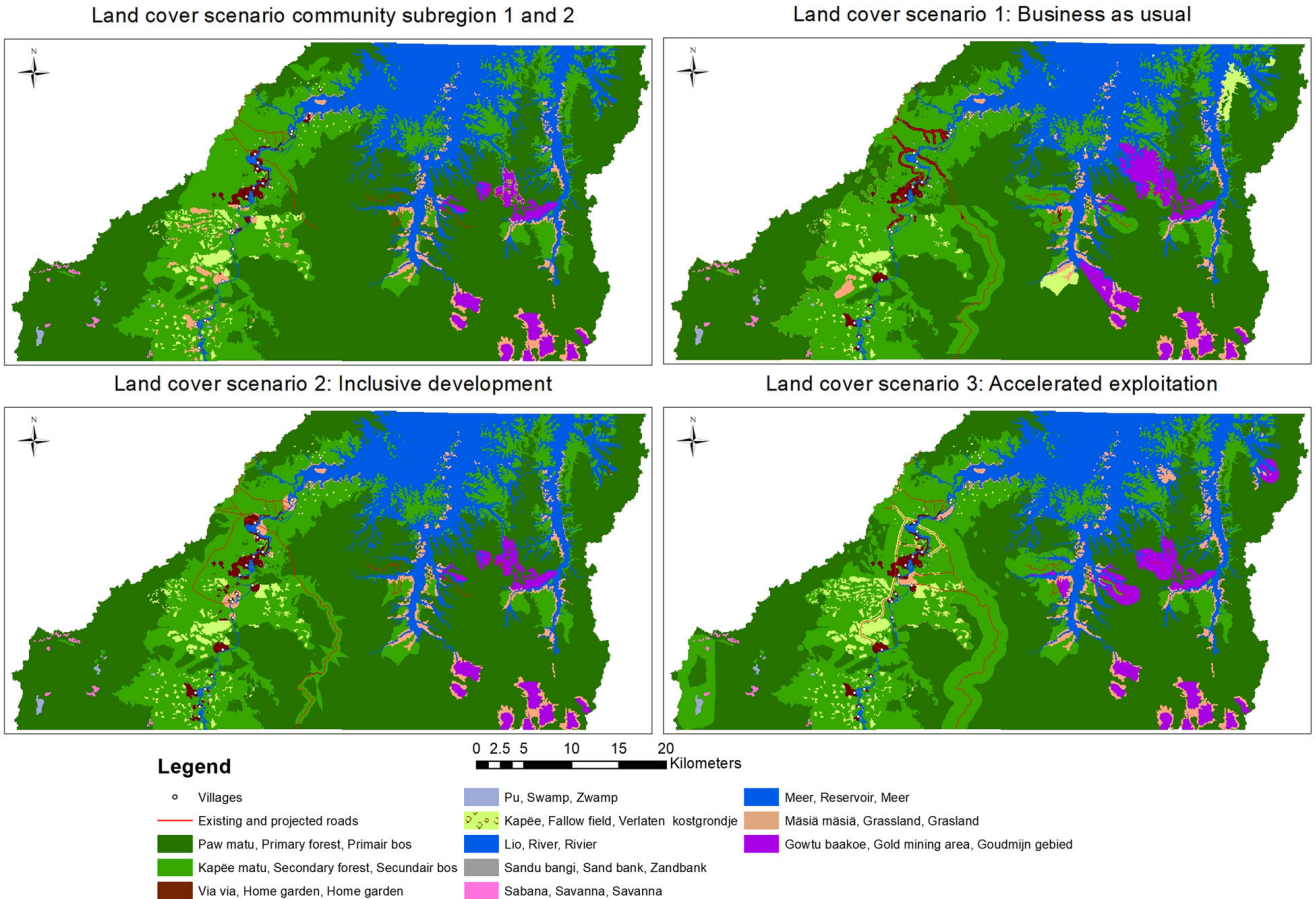
Maps showing changes in land cover for each of the scenario. The upper left map shows the two community scenarios from the respective subregions. The upper right map shows the business-as-usual scenario. The lower left map shows the inclusive development scenario. The lower right map shows the accelerated exploitation scenario. The land cover types match the land covers from the P3DM

The focus group discussions in the subregions resulted in two community scenario narratives with some minor differences. This was expected, as subregion 2 is relatively more isolated. In total, five scenario narratives were drafted (Table 5). The communities’ vision for the future from both subregions had a strong focus on the improvement of basic needs (e.g., opportunities for higher education, 24-h electricity and healthcare), modernization to support livelihoods, and recognition of customary rights and rules related to the territory. The three stakeholder scenarios were: (1) a business-as-usual narrative, (2) an inclusive development narrative, and (3) an accelerated exploitation narrative. Out of the three stakeholder scenarios, the inclusive development narrative most closely resembled the communities’ vision for the future. From the modeling exercise, it is clear that all five scenarios led to some extent of forest degradation, through the expansion of villages, agricultural plots, or logging, or through the development of road infrastructure (Fig. 8). The two community scenarios are shown in the upper left map with the two subregions combined. When it comes to community forest concessions for logging, communities in subregion 1 showed larger areas of primary forests that changed into secondary forests. The inclusive development scenario had less impact when it comes to small-scale goldmining compared to the business-as-usual and the accelerated exploitation scenario.

**Table 5:** Overview of narratives from the participatory scenario planning process

Elements	Demography	Social infrastructure	Economic development	Land use	Governance and organization	Environment
Scenarios						
Community vision subregion 1	Population growth, delayed migration	Improved medical centers, vocational education Permanent electricity	Commercialization and processing of crops and NTFPs with help of technology, increase in minimarkets	Shifting cultivation NTFP harvesting Community forestry Village expansion	Community cooperatives, youth entrepreneurship groups Ban on goldmining, no logging concessions Stricter enforcement of FPIC	Intensification of traditional activities Implications of modernization on solid waste increase
Community vision subregion 2	Population growth, immigration from other areas due to economic activity	Solar energy Waste management system	Commercialization and processing of crops and NTFPs Only locally owned minimarkets allowed Community cooperatives Commercialization of culture for sustainable tourism	Shifting cultivation, NTFP harvesting, Village expansion away from the river Expansion of paths between villages Community forestry (however no new community forest concessions)	Ban on goldmining and no new roads into the area Collaboration with companies in Paramaribo for processing and sale of agro- and timber products Rules and requirements for sustainable tourism Recognition of P3DM map and collective land rights; enforcement remains a challenge	Sustainable use of forest resources
Business-as-usual (stakeholders)	Migration of young people out of the area	Permanent electricity Sub-optimal education, healthcare and employment	More and better stocked mini-markets Increase in tourism Modernization of housing, limited commercialization of customary practices	Two new (paved) roads southwards along the river Increase in land-use conflicts	Collective land rights unresolved. Decentralization of central government, however challenges remain	Degradation of ecosystems, in particular fish and timber
Inclusive development (stakeholders)	Population growth, delayed migration due to employment in tourism for some	Permanent electricity and improved transport of goods and persons Vocational education and medical center at Atjoni	USRB as go-to tourist destination in the interior Urbanization of Atjoni Craft market Improvement of tourism value chain	Village expansion, Recognition of places of archeological importance	Improved community self-organization First regional development plan established in participatory and integrated approach Formalization of FPIC, land rights not yet recognized	Sustainable use of forest resources Challenges with environmental monitoring
Accelerated exploitation (stakeholders)	Delayed migration due to employment in forest exploitation	Increasing inequality due to power structures. Improved facilities for education and healthcare, but quality lacking	USRB, Atjoni, becomes development hub in the Sipaliwini district Optimization of resource exploitation for economic growth	One large community forest concession for the whole USRB Two new roads southwards along the river Intensification of forestry and agriculture	Sustainable forest management plan for community forest concession Decentralization, but still top-down governance Customary governance authorities become symbolic	Increased pollution and degradation of ecosystem services

**Fig. 9:**
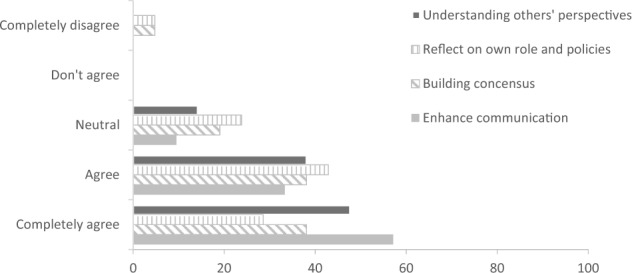
Opinion of stakeholders on the usefulness of participatory scenario planning related to landscape governance

During the final joint workshop with community members and stakeholders, the different scenarios were compared to identify potential synergies, conflicts, and common goals. A brief reflection with the participants indicated that the results needed to be institutionalized and communicated with other community members and stakeholders to increase ownership. In addition, community participants indicated that the PSP process helped them consider potential risks of seemingly positive developments, such as improved accessibility into the area via roads. A survey among participating stakeholders found that almost all participants thought PSP to be useful for enhancing communication between stakeholders and understanding of others’ points of view (Fig. 9). About 75% of respondents agreed that PSP helped to stimulate consensus-building among stakeholders. Furthermore, some of the main perceived benefits of PSP included: (i) gaining new insights into the social-ecological systems, the underlying customs in the landscape, and the potential consequences of land-use decisions; (ii) better involvement and input from local communities; (iii) broader engagement and participation of stakeholders; (iv) enhanced collaboration and “togetherness”, and (v) better consideration of spatial planning aspects. Lastly, respondents noted that more public discussion, in an already time-consuming process, would be necessary to work toward policy changes. Regardless, participants recognized the potential of PSP for spatial planning, sustainable use of forest resources, policymaking, and guidance for developing company projects.

## Discussion

We explored the application of P3DM, the *Trade-off!* game, and PSP in a case study on sound land-use planning and ecosystem services in the USRB. The three tools complemented each other well. First, the P3DM enhanced meaningful engagement with the Saamaka community and it was instrumental for building trust. The resulting map from this process was incorporated in the two other tools. Second, the *Trade-off!* game served as a good ice-breaker with non-local stakeholders and set the stage for further discussion during the PSP. Furthermore, the trade-off concept complemented the PSP since both tools originate from the InVEST toolbox. Hence, the three tools had different but complementary purposes. In the following sections, we discuss the strengths and weaknesses of each of the three tools and show how these, separately and in combination, contribute to inclusive landscape governance.

### Participatory 3-Dimensional Modelling

Four attributes of the P3DM tool were identified as strengths: (i) the participation scheme, (ii) the third dimension of the map, (iii) the large size of the model, and (iv) the blank model. First, the participation scheme enabled discussion between participants from two consecutive clusters, resulting in cross-pollination and a robust triangulation of the data (Rambaldi et al. 2006; Ramírez-Gomez et al. 2017). Second, the bird-eye view and 3D effect of the area enabled a holistic visualization of the entire area, improving accessibility, understanding, and interaction of low- or illiterate women, children, and elders. As shown in similar applications, the 3D view stimulated spontaneous reflection on conflicting landscape interests and the effects on ecosystem services (Gaillard et al. 2013). Third, the large model size enabled better inclusion of many important places across a larger territorial extent. Participants indicated that most maps of the Saamaka territory are restricted to a fringe along the river, excluding many areas of use. The large model size also enabled the co-production of detailed spatial information. The physical model particularly lends itself to represent the communities’ rich, multi-faceted knowledge, values, and interests when communicating and negotiating with stakeholders, thereby contributing to community empowerment (McLain et al. 2013; Zolkafli et al. 2017; Brown and Kyttä 2018). Lastly, the blank model was mapped based entirely on participants’ traditional knowledge. Participants used creeks as initial reference points to locate themselves on the map and translated their tacit social-ecological knowledge onto the model (McCall and Dunn 2012; Needham et al. 2020). The weaknesses of the P3DM tool mainly related to practical aspects, such as the durability of the materials used and ensuring appropriate storage for regular use.

### *Trade-off!* Game

The strengths of the *Trade-off!* game lie in (i) the visual playing boards, (ii) the setup of playing rounds, and (iii) the fictive setting (Verutes and Rosenthal 2014; Verutes et al. 2017; Lasiewicz-Sych 2019). First, the playing boards visualized maps that were adapted to our case study area, making the gaming situation easy to understand and relatable for participants. This stimulated discussion and interaction between group participants, which otherwise may have been less (Verutes and Rosenthal 2014). Second, the setup of the playing rounds stimulated learning among participants. After the first round when participants thought they won, they learned that their playing strategy had also lost them points and that they needed to consider the trade-offs in a new strategy. During the second round, participants included protection of biodiversity and NTFPs in their strategies and learned the potentially larger benefits of land-use decisions when considering the value of ecosystem services. Third, the *Trade-off!* game provided a fictional, safe setting, which stimulated open discussion on land-use strategies (Bellotti et al. 2013; Rodela et al. 2019; Orduña Alegría et al. 2020). The response of the participants was positive, stating that the workshop was “fun and informative”, and that they “better understand the importance of integrating the value of nature in land-use planning”. Some participants even showed interest in playing the game within their respective organizations. Despite its usefulness, the *Trade-off!* game remains a simplified representation of reality (Lasiewicz-Sych 2019) and would require embedding in formal processes to truly prompt changes in practices (e.g. Verutes et al. 2017).

### Participatory Scenario Planning

The strengths of the spatially explicit PSP tool include: (i) the iterative, participatory character, (ii) the model input requirements, and (iii) the visualization of scenario narratives. First, the PSP had several feedback moments with participants, which allowed participants to evaluate their visions for the future, stimulated discussion and consensus-building, and allowed adjustments where necessary (Voinov and Bousquet 2010; McKenzie et al. 2012). This was also perceived by respondents as one of the main advantages of the PSP tool. Overall, the iterative character of the PSP stimulated the consideration of diverse practices, policies, and organizational models for resource use in the USRB (Pacheco et al. 2008; Oteros-Rozas et al. 2015). Second, the Scenario Generator model had simple input requirements, making it useful in a data-scarce context (Ritzema et al. 2010) and for incorporation of participants’ knowledge (Carlsson 2017; Sharma et al. 2018). It was notable how community participants could easily understand the required inputs thanks to their traditional ecological knowledge. Lastly, the visualized scenario narratives helped participants to better understand the interlinkages between systems, processes, and people and to evaluate the implications of their decisions in a spatial sense (Kok et al. 2007; Reed et al. 2013). The main weakness of the PSP is that it can be very time consuming (Berg et al. 2016; Asubonteng et al. 2021 (this issue)), risking stakeholder fatigue. In addition, if the PSP is not institutionalized, it can raise expectations of participants, as concrete outcomes will be limited (Chambers 2006; Verutes et al. 2017).

### Potential for promoting inclusive landscape governance

The relative contributions and limitations of the applied tools are based on criteria of inclusive landscape governance: (i) inclusiveness, (ii) accountability, (iii) equity, (iv) collaboration and coordination, and (v) competence (Table 6). The P3DM contributes most to inclusiveness, equity, and collaboration and coordination. First, by involving different groups, including women and youth, and aiming to empower marginalized communities (Rambaldi 2010). The physical model can support internal governance and strengthen the communities’ voices in landscape management (McCall 2003; McCall and Dunn 2012; Chung et al. 2019). Second, the documentation of tacit knowledge with the physical model and compatibility of P3DM with “modern” GIS legitimizes the communities’ knowledge and gives credibility to the P3DM tool, as also suggested by McCall and Minang (2005), Gaillard et al. (2013), and Ramirez-Gomez et al. (2017). Lastly, the third dimension, the physical size of the model and the detailed spatial information, enabled knowledge sharing and social learning among and within communities (McCall and Dunn 2012; García-Nieto et al. 2019; Akbar et al. 2020). The P3DM contributes relatively less to accountability but did stimulate some transparency in land use within the landscape (McCall and Dunn 2012). Similarly, the P3DM contributes less to competence, but more than to accountability, because it boosts the confidence of community members and is relatively simple to manage compared to more complex geo-information tools (Smith et al. 2017).

**Table 6:** Summary overview of the contribution of each of the applied to tools to landscape governance (+ = strong, +/– = less strong, – = weak)

Criteria based on elements of landscape governance	Inclusiveness Enhancing fair participation, integrating knowledge systems, legitimizing local voices, and tacit knowledge	Accountability Supporting transparent processes, enhancing involvement of actors in all stages	Equity Contributing to empowerment of users and involvement of disadvantaged groups	Collaboration and coordination Stimulating interaction between landscape actors, mutual understanding, social learning. and discussions on trade-offs	Competence Enhancing capacity building for area management, confidence, and accessing information
Strengths of applied tools					
*Participatory 3-Dimensional Modelling*					
Participation scheme	+	+/−	+	−	+
Third dimension	+	−	+	+	+/−
Size of the model	+	−	+	+	−
Populating the blank model	+	−	+/−	+/−	−
*Trade-off! game*					
Visual playing board	+	−	+/−	+	+/−
Setup of playing rounds	−	+/−	−	+	+
Fictive setting	−	−	−	+/−	+/−
*Spatially explicit participatory scenario planning*					
Iterative character	+	+	+	+	+/−
Model input requirements	+	+/−	+/−	+/−	+/−
Visualization of narratives	+/−	+	+/−	+	+

The *Trade-off!* game contributes most to collaboration and coordination and to competence, and least to accountability, equity, and inclusiveness. First, the visual and adaptable playing boards stimulated interaction and discussion between participants that may not have likely have taken place otherwise, contributing to the collective exploration of issues and possible solutions in a landscape (García-Barrios et al. 2008; Speelman et al. 2014; Bosma et al. 2020). Having mixed stakeholder groups could further enhance cross-sector, multi-level discussion and social learning (Voinov and Bousquet 2010; Lasiewicz-Sych 2019). Second, the educational purpose of the *Trade-off!* game enhances capacity building by increasing participants’ knowledge and understanding of land-use trade-offs. The *Trade-off!* game contributes less to inclusiveness, accountability, and equity because its purpose is limited to educating participants, as opposed to, for example, informing interventions and transformative action (Rodela et al. 2019).

The PSP tool contributes most to inclusiveness, collaboration and coordination, and accountability. First, jointly discussing the shared future of the landscape enhances communication between landscape actors and the integration of different types of knowledge (Heemskerk 2003; Patel et al. 2007). The PSP tool created a space where indigenous and tribal communities can represent themselves and voice their interests (Bou Nassar et al. 2020). Second, the iterative interaction and reflection between diverse landscape actors enhance mutual understanding, social learning, and negotiating trade-offs. Furthermore, the PSP stimulates transparency by involving different actors at all stages and discussing the roles and responsibilities in the scenarios, in particular when PSP is integrated in formal processes (Saah et al. 2019; Carnohan et al. 2020). The PSP tool contributes less to equity and competence, although the community scenarios present a potential instrument to represent their values and preferences toward a self-determined future (Chung et al. 2019; Needham et al. 2020).

When comparing the three tools applied in our case study to the many other existing geo-information tools (Kozar et al. 2014; Chung et al. 2019; Akbar et al. 2020; Flacke et al. 2020), we cannot simply state that one is better than the other. Our case study included participants with a strong business-as-usual rationale and marginalized, low-literate communities with a history of distrust toward external actors, which all were considered in developing our approach. With the availability of so many participatory geo-information tools, it is important to carefully consider how the strengths and weaknesses of a tool relate to the local reality and set aims. Moreover, we argue that consideration should be given to how tools may complement each other and amplify their strengths, as found in our case study. First, a combination of tools builds momentum and understanding among a diverse group of landscape actors. In addition, complementary tools provide detailed insights into multi-dimensional aspects of landscape dynamics and inclusive governance. Furthermore, combining tools helps to identify interlinkages between specific issues and work toward solutions in an integrated manner. As such, there is added value in strategically applying a combination of participatory geo-information tools.

### Limitations of Our Case Study

The completion of P3DM is not an endpoint in itself, rather the beginning of a process. Much of its success depends on the process of facilitation (Chambers 2006; Voinov and Bousquet 2010; Bou Nassar et al. 2020) and participation (Maceda et al. 2009; Gaillard et al. 2013). A follow-up stage is necessary, including updates to the model, as circumstances and communities’ social-ecological understanding changes (McLain et al. 2013). Optimal use of P3DM may still require guidance and additional capacity strengthening (Maceda et al. 2009; Smith et al. 2017), especially for more vulnerable groups such as women and youth. Although many women were present during the legend workshop, which led to an extensive list of items important to female community members, the availability of women during the longer mapping phase was sometimes limited. This was due to their traditional gender roles and related responsibilities. As such, the areas shown on the map that are important to women’s livelihood are not exhaustive. Another limitation is the rightful fear among communities that the resulting spatial information could be used against them, stressing the importance to discuss and reach consensus on data ownership and permission issues upfront (Chambers 2006; McLain et al. 2013; Brown and Kyttä 2018; Wheeler and Root-Bernstein 2020). Using principles of free, prior, and informed consent can keep agreements transparent and hold facilitating organizations accountable, although it may not completely avoid information misuse.

With the *Trade-off!* game there was a stronger focus on adapting and applying it, than on evaluating learning outcomes. In part, the capacity for facilitating this evaluation was lacking from the side of the research team. While literature suggests that serious games stimulate learning (Ricci et al. 1996; Kiili 2007; Webb et al. 2012; Bellotti et al. 2013), methods for the systematic evaluation of the effectiveness of games are often lacking (Bellotti et al. 2013; Speelman et al. 2017; van Noordwijk et al. 2020). Systematically evaluating the effectiveness of the *Trade-off!* game would provide valuable insights into landscape actors’ worldview and the decisions they make.

Another limitation was that the scenarios were not validated beyond the group of participating community members and stakeholders, due to the scope of the case study and limited resources. Broad community support and ownership are necessary for the PSP to be successful in formal planning processes. In addition, participants were selected based on self-determination principles, which is a sign of respect toward the Saamaka community. However, this can also lead to exclusion of families or persons due to internal power relations.

Lastly, general factors to be considered to optimally use the three tools include (i) excellent facilitation, which strongly affects the process; (ii) a well-thought through participation scheme, based on appropriate and respectful approaches, to avoid selection bias and aggravating internal power struggles; (iii) addressing sensitive information and data ownership issues; (iv) clarifying the scope and underlying assumptions to not raise expectations; and (v) institutionalization of tools and outputs in formal processes to enhance fair influence of participants in landscape management (McLain et al. 2013).

## Conclusion

We explored the application of three spatially explicit, participatory tools in a contested tropical forest landscape with marginalized communities and customary institutions. The P3DM tool, the *Trade-off!* game, and the PSP tool complemented each other well. All three tools draw their strengths from their visual and highly interactive characteristics, making them more accessible to disadvantaged and low-literate persons and stimulating social learning. The three-dimensional attribute of the P3DM tool enabled a holistic visualization of the entire landscape, which was effective to remove epistemological barriers between researchers, governmental stakeholders, and local community participants. Reflecting on the strengths of the three spatially explicit, participatory tools against criteria for inclusive landscape governance, we found that the tools help to enhance various aspects of inclusive landscape governance. Moreover, the complementarity of the applied tools proved to be of added value and is worth assessing in similar landscapes.

Further research could focus on answering the question how to best select participatory geo-information tools or a combination of tools in relation to specific objectives and local realities. In addition, there is a need for further elaboration of frameworks to systematically evaluate the effectiveness of participatory, geo-information tools, and combinations of tools. Not just for enhancing inclusiveness and social learning, but also in terms of “adoptability” in formal processes. Finally, further research could assess which combinations of type of tools may fit best. That way, geo-information tools will not only result in spatial information, but can be strategically used (e.g., in action research) to contribute to inclusive landscape governance.

## Supplementary information

Online Resource 1A-C

## Footnotes

1 More details of this event can be found in the blog: Being on a map means to exist: the Saramaccan experience http://www.cta.int/en/article/2016-03-08/saramacca-communities-in-suriname-seek-governmentrs-recognition-of-theirtraditional-knowledge.html.
