# Supplementary material for: Toward Inclusive Landscape Governance in Contested Landscapes: Exploring the Contribution of Participatory Tools in the Upper Suriname River Basin
Source: Environ Manage. 2021 Aug 10;68(5):683–700. doi: 10.1007/s00267-021-01504-8 (PMC8560725; doi:10.1007/s00267-021-01504-8)
Supplement: Supplementary file 1 — Online Resource 1A-C [file 267_2021_1504_MOESM1_ESM.docx]

## **ONLINE RESOURCE 1A-C**

## **Toward inclusive landscape governance in contested landscapes: exploring the contribution of participatory tools in the Upper Suriname River Basin**

Best, L.^a,b*^, Fung-Loy, K.^c^, Ilahibaks, N.^a^, Ramirez-Gomez, S.^a^, Speelman, E.N.^b^

^a^ Tropenbos Suriname. Prof. Dr. Ruinardlaan (University campus), CELOS building. P.O.Box 4194. Paramaribo, Suriname.

^b^ Laboratory of Geo-information Science and Remote Sensing, Wageningen University & Research, 6708 PB Wageningen, The Netherlands.

^c^ Anton de Kom University of Suriname. Department of Sustainable Management of Natural Resources. P.O.Box 9212. Paramaribo, Suriname.

^*^ Corresponding author: l.best@tropenbos.sr

ORCID:

Best, L.: https://orcid.org/0000-0003-1122-8767

Fung-Loy, K.: https://orcid.org/0000-0002-4161-3925

Ilahibaks, N.: https://orcid.org/0000-0002-9853-8540

Ramirez, S.: https://orcid.org/0000-0002-7081-5550

Speelman, E.N.: https://orcid.org/0000-0003-1842-4939

1. **Detailed methodology of the Participatory 3-Dimensional Modelling**

Selecting participants:

- A ‘profile’ of participants was explained to the community: school children for the first phase of constructing the model, women and men who have tacit knowledge about the area, as well as older community members who have historical knowledge to add spatial information to the model. From the P3DM process a core group of participants formed itself. The same group also participated in the PSP process.
- Participants outside of the landscape were selected based on an initial analysis of stakeholders for the USRB that included public, private, civil society and academic sectors, and community representative organizations. Participants were first approached for the Trade-off! game and later on for the PSP process.

Participation scheme:

The participation of local community members was organized in 4-5 clusters of villages in each subregion allowing free and open deliberations between neighboring villages about landscape services, values, preferences and drivers of change. The time planning included a brief overlap of consecutive clusters.

- Developing the map legend - Focus group discussions with in total 110 community members (80 men and 30 women).
- Constructing and populating the model - Two mapping workshops with in total 105 community members (84 men and 21 women).
- Validation of the P3DM maps - Six validation workshops with in total 100 community members (78 men and 22 women).
- Presentation and reflection – presentation event with 32 external stakeholders.

Dimensions of the models: The P3DM in the two subregions consisted of two models of 5 m x 2.5 m each, at a scale of 1:15000.

1. **Detailed methodology of the Trade-off! game**

Selecting participants:

Stakeholders were identified following a brief analysis during the preparatory phase and invited to self-select a representative to attend the gaming workshop.

Adapting the game:

- The boards were based on the digitized GIS data obtained from the P3DM: line data (roads), point data (agricultural plots, tourist lodges, NTFP hotspots), polygon data (river rapids, high biodiversity areas). A 1x1 km^2^ grid was used to categorize the points that could be gained, where cells closer to the developments or ecosystem services would be worth more points than those further off. The points used in the grid cells are relative (125, 100, 75, 50, and 25, with darker colored cells worth more than lighter colored ones) and meant only for the purpose of determining the winner of the game.
- The fourth map showed the potential points to be lost due to damages to ecosystem services. The ecosystem services considered were Biodiversity (habitat) and provision of NTFPs. As there was no biodiversity data available at the time, the biodiversity map was based on hypothetical hotspots created for the exercise, showing areas of higher, medium, and lower biodiversity (125, 100, and 75 points respectively). NTFP provision was shown on this map as a single colored point, with each point being worth 125 points. The locations for the NTFP’s were obtained from the P3DM. As the maps were adapted for Suriname, the original online score calculator had to be adapted in MS Excel, corresponding to the maps.

Playing rounds:

- In the first round, the groups are asked to develop the area by placing a number of ‘development’ pawns in the grid cells on the playing board to try and maximize their total points: 60 pawns for infrastructure, 30 for agriculture and 10 for tourism. Two pawns on different maps could not be placed on the same grid cell and road infrastructure pawns had to be logically connected.
- At the end of the first round, the scores are calculated per group by adding the points gained from all of the development maps, taking into account each used grid cell, as well as its eight adjacent cells. This simulates the positive impact development in a certain location can have on its surroundings. The calculation of the scores was done by manually entering the locations of the development pawns in the corresponding maps in an Excel calculator.
- At the beginning of round 2, participants are introduced to the concept of ecosystem services. The calculator is used to reveal that each group also lost points and participants learn that their development decisions in the first round had a negative impact on local ecosystem services. A new, net score is calculated from the impact of the developments placed in round 1 by subtracting the points lost from the points gained and shown to the groups.
- To play the second round, the fourth map with ecosystem services is added so participants can see the locations where developments would have higher negative impacts than others. The playing objective of participants in this round was to maximize economic gains from development, while trying to minimize the harm to ecosystem services, depending on the location that would be developed. The groups can move their development pawns to a new location or use ‘protection’ pawns, 50 pieces. By placing protection pawns in a grid cell, points from the protected cell are neither lost nor gained. The round 2 score is then calculated (Fig. B1). The ‘developments’ and ‘protection’ pawns on the maps were entered manually into the corresponding maps in Excel.


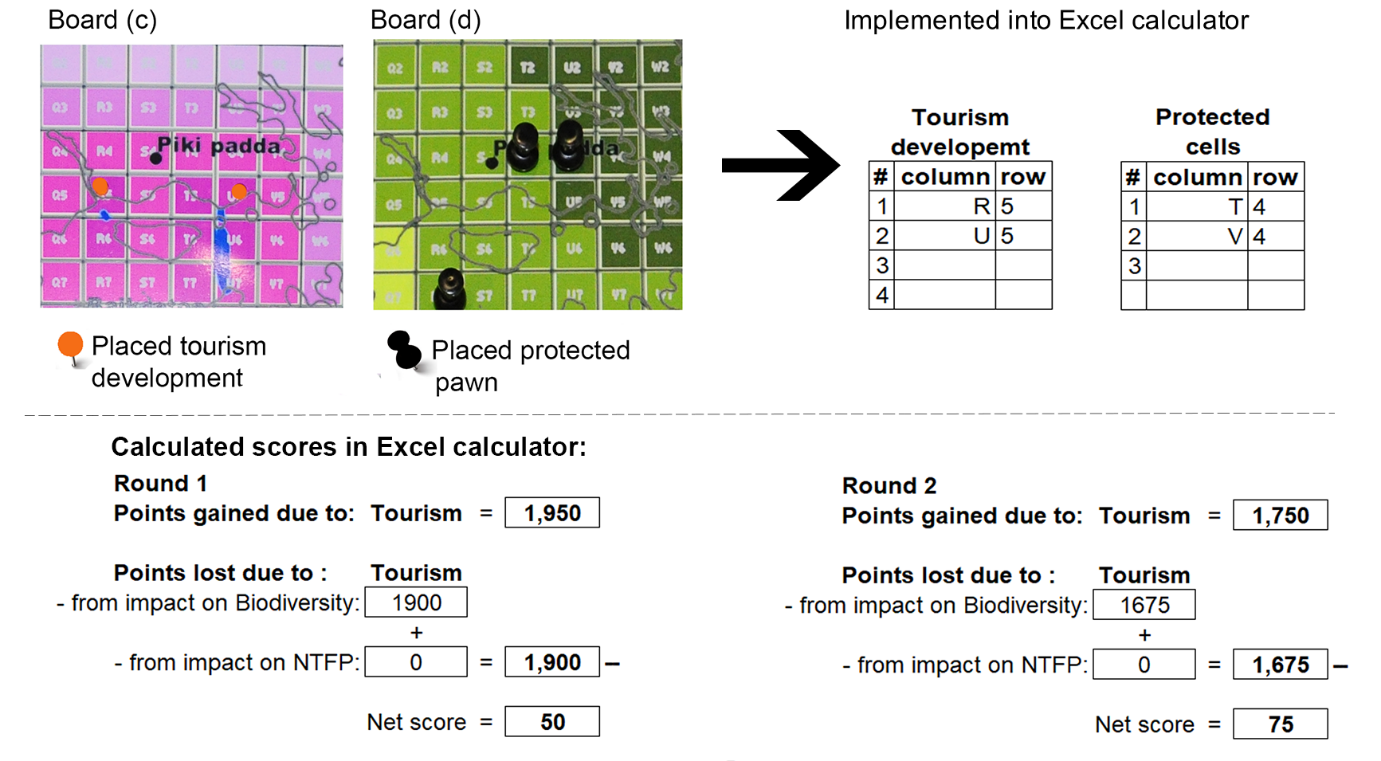


**Fig. B1** An example of score calculation in the Excel calculator. Tourism development placed on board (c) would gain 1950 points and lose 1900 points in round 1. In round 2, as a result of placing ‘protection’ pawns, on board (d), less points are gained (1750) but also less points are lost (1675)

1. **Detailed methodology of the Participatory Scenario Planning**

Setting the scope and identifying stakeholders:

During the first phase the purpose and desired outcomes within the context of the project (why do scenario development, what important outputs, including learning) were defined by the research team to set the boundaries and scope for this part of the project. In addition, stakeholders were identified during a preparatory session with the research team, based on different sectors (e.g. forestry, agriculture), types (e.g. government, civil society, community and community-based) and interests of stakeholders in the landscape.

Gathering information for the narratives:

During the second phase questions and methods were prepared for two groups: the Saamaka community residing in the landscape and stakeholders of the area residing outside the landscape. For the community members, two-day sessions were held in each of the two subregions. Focus group discussions were conducted using visual aids, including the digitized P3DM map and supporting discussion questions, to allow active participation of any illiterate or low literate community members. Based on the discussion, a narrative was formulated. Furthermore, land cover transitions and suitability factors were discussed with participants based on the drivers of change identified in the narrative. The land cover classes used were aligned with the ones constructed during the P3DM process by the community themselves. For the stakeholders in Paramaribo, which formed a more diverse group, seventeen semi-structured interviews were conducted with representatives from different sectors. Based on the interview responses, three narratives were drafted.

Drafting and validating the narratives:

During the third phase, the narratives were drafted and the input on land cover transition probabilities and land suitability factors was gathered in a follow-up session with community members and stakeholders.

The fourth phase comprised of modelling the spatially explicit scenario’s and discussing this in a feedback session to further fine-tune the narratives, land cover transitions and land suitability factors.

Presentation and reflection:

During the fifth and sixth phase, the scenarios and associated maps were finalized and presented to stakeholders and community participants during a second, plenary workshop, bringing all actors together to reflect upon the differences and similarities.
